# Cenderitide, a novel dual GC-A and GC-B receptor activator, is a potent chronic cardiorenal fibroinhibiting peptide which suppresses aldosterone and reduces proteinuria in models of ardiorenal fibrosis

**DOI:** 10.1186/1471-2210-11-S1-P22

**Published:** 2011-08-01

**Authors:** Fernando L Martin, Paul M McKie, Jeson S Sangaralingham, Tomoko Ichiki, Gerald E Harders, Horng H Chen, John C Burnett

**Affiliations:** 1 Cardiorenal Research Laboratory, Mayo Clinic, Rochester, MN, USA

## Background

Cenderitide, also known as CD-NP, is a novel Mayo engineered designer natriuretic peptide (NP), which unlike native ANP, BNP and CNP co-activates both the guanylyl (GC)-A receptor to which ANP and BNP bind and GC-B to which CNP binds. Recognizing the aldosterone suppressing actions of GC-A activation and the potent inhibition of collagen synthesis and proliferation on fibroblasts to GC-B activation we hypothesized cenderitide would be a potent anti-fibrotic therapeutic agent. Here we defined the actions of chronic cenderitide administrated subcutaneously by pump in two models of cardiorenal fibrosis which included in a model of post myocardial infarction (MI) induced cardiorenal fibrosis and also in a model of chronic kidney disease produced by uninephrectomy (UNX).

## Methods

Cenderitide was administered for 2 weeks (170 ng/kg/min, Nile Therapeutics) with an osmotic pump following MI (n=10) or UNX (n=10) while the control groups received vehicle. Cardiorenal function and structure were assessed 3 to 4 weeks after MI or UNX.

## Results

Cardiorenal fibrosis was markedly suppressed by cenderitide in both models of fibrosis. Specifically, following MI, collagen content was decreased in the LV (MI:5±0.6, CD-NP:3.5±0.4 %, p<0.05), and also in the renal cortex and medulla (MI:3.5±0.5, CD-NP:1.3±0.3, p=0.002 and MI:19±5 vs CD-NP:1.2±0.3 %, p=0.0006). After UNX, CD-NP suppressed LV fibrosis vs. UNX (UNX: 4.2±0.5, CD-NP:3±0.4%, p<0.05) and reduced medullary fibrosis. CD-NP reduced aldosterone in the MI group (MI:39.4±10, CDNP: 15±1 dl/ml, p=0.036) as well in the UNX group (UNX: 30.4±6, CD-NP: 12.6±1.8 ng/dL, p<0.05). Importantly, proteinuria was also significantly reduced in CD-NP (p<0.001) in both the MI and UNX group.

## Conclusion

Cenderitide is a novel designer NP that acts as a potent cardiorenal fibro-inhibiting therapeutic agent. It inhibits cardiorenal fibrosis, suppresses aldosterone and reduces proteinuria in two models of cardiorenal fibrosis. These studies support chronic SQ CD-NP as an innovative anti-fibrotic therapeutic.

